# A study on the identification of wheat powdery mildew using chlorophyll fluorescence imaging

**DOI:** 10.3389/fpls.2026.1837843

**Published:** 2026-10-12

**Authors:** Yijia Chen, Chengyuan Hao, Zhiyu Li, Xupeng An, Jingxiang Huang, Xinchang Chen, Shidong Wang, Zhichao Chen

**Affiliations:** 1 School of Geomatics and Aerospace Information, Henan Polytechnic University, Jiaozuo, China; 2 Observation and Research Station of Land Ecology and Land Use in the Grain-Producing Area of Central China, Ministry of Natural Resources, Jiaozuo, China

**Keywords:** chlorophyll fluorescence imaging, deep learning, image classification, powdery mildew of wheat, smart agriculture

## Abstract

**Introduction:**

Wheat powdery mildew poses a serious threat to global wheat production, and its accurate identification is key to smart agriculture.

**Methods:**

This study combines chlorophyll fluorescence imaging with deep learning algorithms to investigate the temporal dynamics of key fluorescence parameters (such as maximum photochemical efficiency, QYmax) during the progression of powderymildew. A QYmax-based chlorophyll fluorescence image dataset for wheat powdery mildew was constructed. Building on this, the LCRNet model was proposed, which effectively processes fluorescence image data through a collaborative mechanism integrating the Large Selective Adaptive Fusion (LSAF) and Coordinated Attention (CA) modules.

**Results:**

On an independent test set, the model achieved an accuracy of 94% and an F1 score of 94.1%, outperforming comparison models.

**Discussion:**

This study proposes a method for classifying the severity of powdery mildew in wheat that reduces the computational cost of fluorescence data processing, providing important methodological support and technical insights for advancing the intelligent, precise, and green transformation of crop disease prevention and control systems.

## 1 Introduction

Wheat is one of the world’s most important food crops and accounts for approximately 20% of global human protein and caloric intake. Its yield stability is directly linked to food security and economic returns (Reynolds and Braun, 2022; Gupta et al., 2024; Sharma and Sharma, 2025). However, wheat growth is highly susceptible to various diseases, which have become key constraints affecting wheat production. Among these, wheat powdery mildew caused by *Blumeria graminis f.* sp. *tritici (Bgt)* can lead to severe yield reductions, with losses reaching up to 50% under extreme conditions (Ashmawy et al., 2014). Due to its high genetic variability, environmental adaptability, and rapid evolutionary rate, this disease readily overcomes host resistance and compromises control measures (Cowger et al., 2016; Jigisha et al., 2025; Latorre, 2025). The infection initiates when ascospores germinate and penetrate the epidermal cells of wheat leaves under humid conditions, subsequently forming colonies and producing conidia that gradually spread across the leaf surface. At the onset of infection, scattered white powdery spots appear on the abaxial leaf surface, followed by the gradual enlargement and spread of the powdery patches (Jankovics et al., 2015; Mapuranga et al., 2022; Önler and Köycü, 2024). The disease thrives under temperatures of 15-22 °C and relative humidity exceeding 70%, with an incubation period of approximately 7 days (Kayim et al., 2022; Simpfendorfer et al., 2023). Therefore, identification and timely management are crucial for containing the spread of powdery mildew.

Traditional methods for detecting wheat powdery mildew primarily rely on expert experience, manual observation, and laboratory sample identification (Singh et al., 2020; Zang et al., 2025). However, these approaches are time-consuming, labor-intensive, costly, and highly subjective, rendering them inadequate for the requirements of contemporary precision agriculture (Xuan et al., 2022; Song et al., 2026). To overcome this bottleneck, Chlorophyll Fluorescence Imaging has emerged. This technology offers advantages such as non-invasiveness, high sensitivity, and real-time monitoring capabilities. It effectively addresses the shortcomings of traditional methods—including time-consuming procedures, reliance on obvious symptoms, and difficulty in quantifying dynamic changes—and has evolved into a crucial cutting-edge diagnostic tool in plant physiology research (Ivanov and Bernards, 2016; Méline et al., 2020; Moustakas et al., 2021; Moustaka and Moustakas, 2023). By monitoring the intensity and kinetic characteristics of fluorescence signals emitted by chlorophyll after absorbing light energy, this technique enables non-destructive assessment of photosynthetic efficiency. Its principle is grounded in the competitive allocation of light energy during plant photosynthesis: absorbed light energy is primarily distributed through three pathways—photochemistry, heat dissipation and chlorophyll fluorescence emission—which maintain a dynamic equilibrium where one increases as the others decrease (Porcar-Castell et al., 2014; Ma et al., 2025). Consequently, changes in fluorescence signals can reflect the light energy conversion efficiency of Photosystem II (PSII) in real time, enabling precise assessment of plant photosynthetic physiology and responses to environmental stress. This technology is widely applied to crops such as rice, tomatoes, and strawberries (Šebela et al., 2018; Pineda et al., 2018; Meng et al., 2020; Stamelou et al., 2021; Suárez et al., 2022; Sun et al., 2023; Chen et al., 2024; Lee et al., 2025). Chlorophyll fluorescence indices, as key physiological parameters characterizing photosynthetic status, provide highly targeted means for detecting plant stress responses. QYmax (Fv/Fm) represents the maximum photochemical efficiency of PSII, and its decline reflects the extent of photosystem damage (Maxwell and Johnson, 2000; Baker, 2008). The fluorescence decay rate Rfd (Fd/Fs) has been proposed as a plant “vitality index” to characterize leaf photosynthetic activity (Lichtenthaler et al., 1986; Ptushenko et al., 2014). In studies on the light-induced greening of etiolated barley leaves, Rfd values have increased significantly in the middle and lower leaf regions of etiolated barley seedlings after 10–12 hours of light exposure, a result that further supports its effectiveness in assessing photosynthetic potential (Babani and Lichtenthaler, 1996). Y(NO) describes the quantum yield of non-regulated heat dissipation, reflecting the degree of irreversible photoinhibition caused by excess excitation energy not being effectively dissipated (Kramer et al., 2004). These fluorescence parameters are critical for the precise grading of disease severity in wheat. However, practical field application of this technology still faces challenges such as natural environmental fluctuations, high equipment costs, and operational complexity, which constrain its full potential for early warning (Murchie and Lawson, 2013; Kalaji et al., 2014; Kalaji et al., 2017). Therefore, efforts are underway to integrate chlorophyll fluorescence imaging with intelligent analysis techniques like machine learning and deep learning to achieve breakthroughs.

In recent years, the integration of chlorophyll fluorescence imaging with machine learning and deep learning has provided more robust technical support for the diagnosis of plant stress, with its value demonstrated in multiple studies. Zhang et al. (2025) have employed a 2D-CNN model driven by multispectral-chlorophyll fluorescence fusion images for the classification of Verticillium wilt in egg-plant, achieving a classification accuracy of 99.37% and successfully detecting disease symptoms as soon as the third day post-inoculation (Zhang et al., 2025). Cen et al. (2017) have extracted fluorescence parameters and image features from citrus leaves using chlorophyll fluorescence imaging. After feature selection and fusion, these features have been input into a classifier, achieving high-precision discrimination among healthy, Huanglongbing (HLB)-infected, and nutrient-deficient leaves, with an accuracy of 97% (Cen et al., 2017). Yang et al. (2025) have employed chlorophyll fluorescence imaging to detect ChlF heterogeneity during the asymptomatic phase of cotton verticillium wilt, achieving pixel-level inversion of ChlF heterogeneity features. This study provides a novel methodology for early non-destructive detection of cotton verticillium wilt (Yang et al., 2025). The versatility of deep learning in agricultural vision tasks is further exemplified by its application in other areas, such as detecting the growth stages of crops like strawberries (Li et al., 2024), and lightweight object detection in remote sensing imagery (Li et al., 2026). Although the machine-learning and deep-learning methods applied in these studies offer high-precision solutions for plant disease identification, they still face challenges such as data imbalance and mislabeling, and overfitting with small sample settings (Li et al., 2024; Zhao et al., 2025). These challenges are particularly pronounced in specialized datasets constructed for wheat powdery mildew fluorescence images. Therefore, it is imperative to develop a method tailored for wheat powdery mildew severity classification using fluorescence images as input.

To address these challenges, this paper proposes a residual network model named LCRNet (a convolutional model for large-scale kernel selection based on residual structures). It is designed to reduce the processing cost of fluorescence data in wheat powdery mildew recognition. The core innovation of this model lies in the design of an efficient feature processing module. This module expands the effective receptive field by introducing the Large Selective Adaptive Fusion (LSAF) module and enhances feature fusion capabilities through the Coordinate Attention (CA) module, thereby achieving efficient processing of chlorophyll fluorescence features and optimized recognition accuracy. Specifically, the LSAF module achieves adaptive feature fusion through multi-branch convolutions and content-driven attention mechanisms. By decomposing large-kernel convolutions into a series of depth-wise convolutions with incrementally expanding dilation rates, it enhances the feature extraction efficiency from fluorescence images (Li et al., 2023). The CA module, functioning as a plug-and-play unit, effectively boosts feature discriminative power by applying globally informed weighting to each channel of feature maps (Hou et al., 2021). Based on the LSAF-CA synergistic mechanism, the proposed LCRNet model maintains a balance between computational efficiency and accuracy, offering an approach for crop disease classification.

The remainder of this paper is organized as follows: Section 2 introduces the materials and methods; Section 3 presents the experimental analysis; Section 4 presents the results; Section 5 discusses the findings; and Section 6 summarizes the article.

## 2 Materials and methods

### 2.1 Data acquisition

This study has been conducted at the experimental field of the Jiaozuo Academy of Agricultural and Forestry Sciences in Henan Province (35.23° N,113.28° E). Under natural field conditions of wheat powdery mildew occurrence, chlorophyll fluorescence parameters and their corresponding high-resolution images have been systematically collected. To control spatial heterogeneity and facilitate measurements, the experimental field has been uniformly divided into eight plots to observe powdery-mildew development in wheat. Within each plot, four sampling transects have been established. To minimize sampling bias, one wheat plant has been randomly selected from each transect. The third leaf (counting from the top) of each selected plant has been used as the measurement site. This leaf position, being a key functional leaf, is highly sensitive to disease onset and represents a typical state of disease progression. Therefore, it has been uniformly adopted as the sampling site across all plants. For each measurement, a new leaf at the same specified leaf position has been sampled from within the plot. Data has been collected during the heading, flowering, and grain-filling stages of wheat. All collections have been performed under consistent field management practices to ensure the natural occurrence and progression of the disease.

Specifically, professional chlorophyll fluorescence imaging equipment has been used for data acquisition. The imaging system employed has been the Flat Handy FluorCam FC 1300-H. This system consists of a main unit, a leaf clip, manually adjustable stage, and the accompanying FluorCam 10 software. The main unit incorporates a high-resolution TOMI-2 CCD camera (1360 × 1024 pixels,16-bit A/D conversion, spectral range 400–1000 nm) which, when paired with dedicated filter components (detection band 695–770 nm), enables highly sensitive capture of chlorophyll fluorescence signals. For excitation, the system utilizes four sets of high-brightness LED arrays, including red-orange (610–630 nm) and far-red (710–740 nm) light sources. These light sources enable the system to deliver both brief, high-intensity saturation pulses (*>* 4500*µ*mol m^−2^ s^−1^) to induce maximum fluorescence (Fm) and sustained, moderate-intensity actinic illumination (up to 1200 *µ*mol m^−2^ s^−1^) for monitoring photochemical kinetic processes. To minimize environmental interference, all field measurements have been conducted under conditions avoiding direct sunlight. Prior to imaging, the target leaves have been clamped and dark-adapted for 15 minutes to ensure reliable acquisition of the baseline fluorescence (Fo) signal. The imaging system has been connected to a computer via Ethernet interface, with FluorCam 10 software controlling the image acquisition and recording fluorescence image series. In this study, the following fluorescence parameters have been primarily acquired and analyzed: QYmax (Fv/Fm), Rfd and Y(NO). Manual photography has been used primarily to obtain data for disease severity assessment. A photograph of the instrumentation is shown in **Figure 1**.

**Figure 1:**
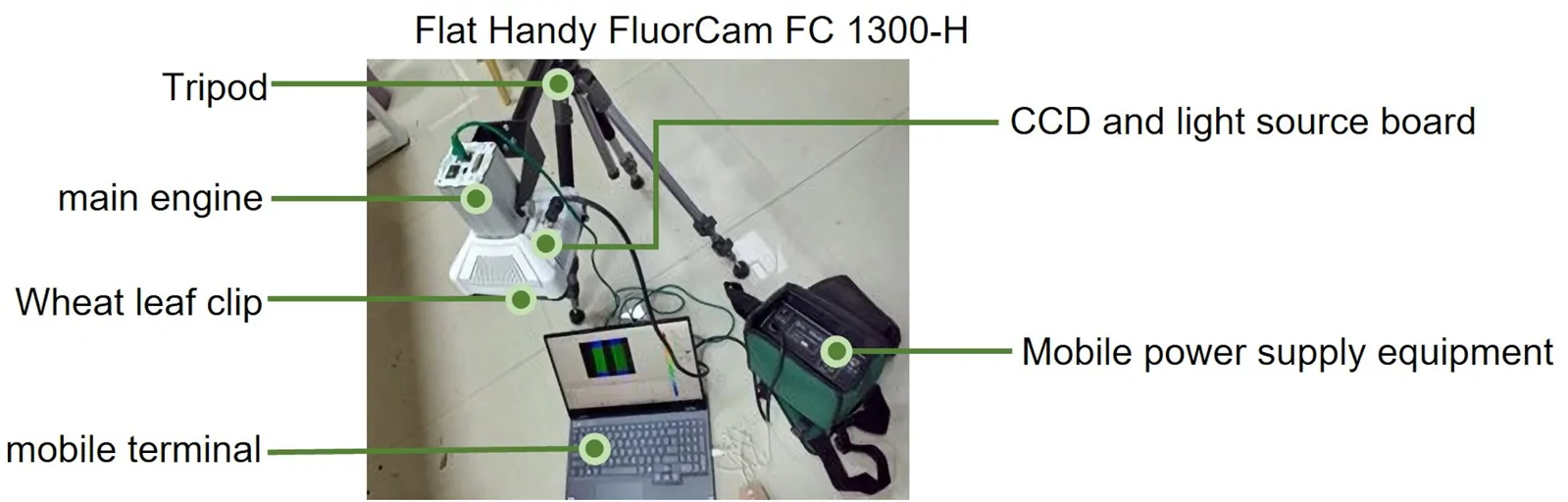
Equipment diagram of the FluorCam portable chlorophyll fluorescence imaging instrument. Key specifications are as follows: Tripod: maximum measurement height of 1.5 m, suitable for fixed-height and field measurements; Main engine: dimensions 194 × 182 × 280 mm, weight 2 kg; CCD and light source plate: provides actinic light and captures fluorescent images; Wheat leaf clip: designed for dark adaptation measurements; Mobile terminal: runs on a 64-bit Windows 10 operating system; Mobile power supply: endurance ≥ 10 h. Key optical parameters: CCD resolution 1360 × 1024 pixels (16-bit), spectral range 400–1000 nm, imaging area 40 × 40 mm; measurement light at 620 nm; photochemical/saturation light at 440–450 nm (max intensity 4500 *µ*mol m^−2^ s^−1^); far-red light at 735 nm. Output parameters: includes *F_o_*, *F_m_*, *QY_max_*, Φ*_PSII_*, NPQ, ETR, and over 50 other chlorophyll fluorescence parameters, as well as GFP distribution map.

### 2.2 Data processing

A total of 96 wheat leaves have been collected, from which chlorophyll fluorescence images and related parameters have been obtained. Due to data anomalies in some samples from the initial collection in Plot 6, two samples have been excluded based on quality control, resulting in 94 valid samples for subsequent analysis. The acquired fluorescence image data have been processed and analyzed using the instrument’s dedicated software, FluorCam 10. First, in the Result interface, regions of interest (ROIs) have been manually delineated using the Selection tool to remove background signals, and each sample has been renamed. Subsequently, the standard analysis protocol has been executed within the Analyst module, where the software automatically calculates parameters and displays the results back in the Result interface. To ensure comparability across samples, the pseudo-color scales for all fluorescence parameter images have been normalized to a common range of 0 to 1 prior to analysis. Finally, the fluorescence data (including QYmax (Fv/Fm), Rfd, Y(NO), etc.) have been exported in Excel format, along with the corresponding fluorescence images. To obtain the disease severity (DS) index, leaf images collected on the same day were imported into the Leaf Doctor software for analysis. The reliability of Leaf Doctor in disease severity assessment has been previously validated (Pethybridge and Nelson, 2015). Subsequent comparative studies have further reported significant positive correlations between Leaf Doctor-based measurements and visual assessments across different leaf spot diseases (r = 0.664–0.899) (Alheeti et al., 2021). The results are presented in **Figure 2**. This software automatically identifies diseased leaf areas and calculates the proportion of lesion area relative to the total leaf area, thereby quantifying disease severity. In this study, disease severity is expressed as the percentage of leaf area affected (%).

**Figure 2:**
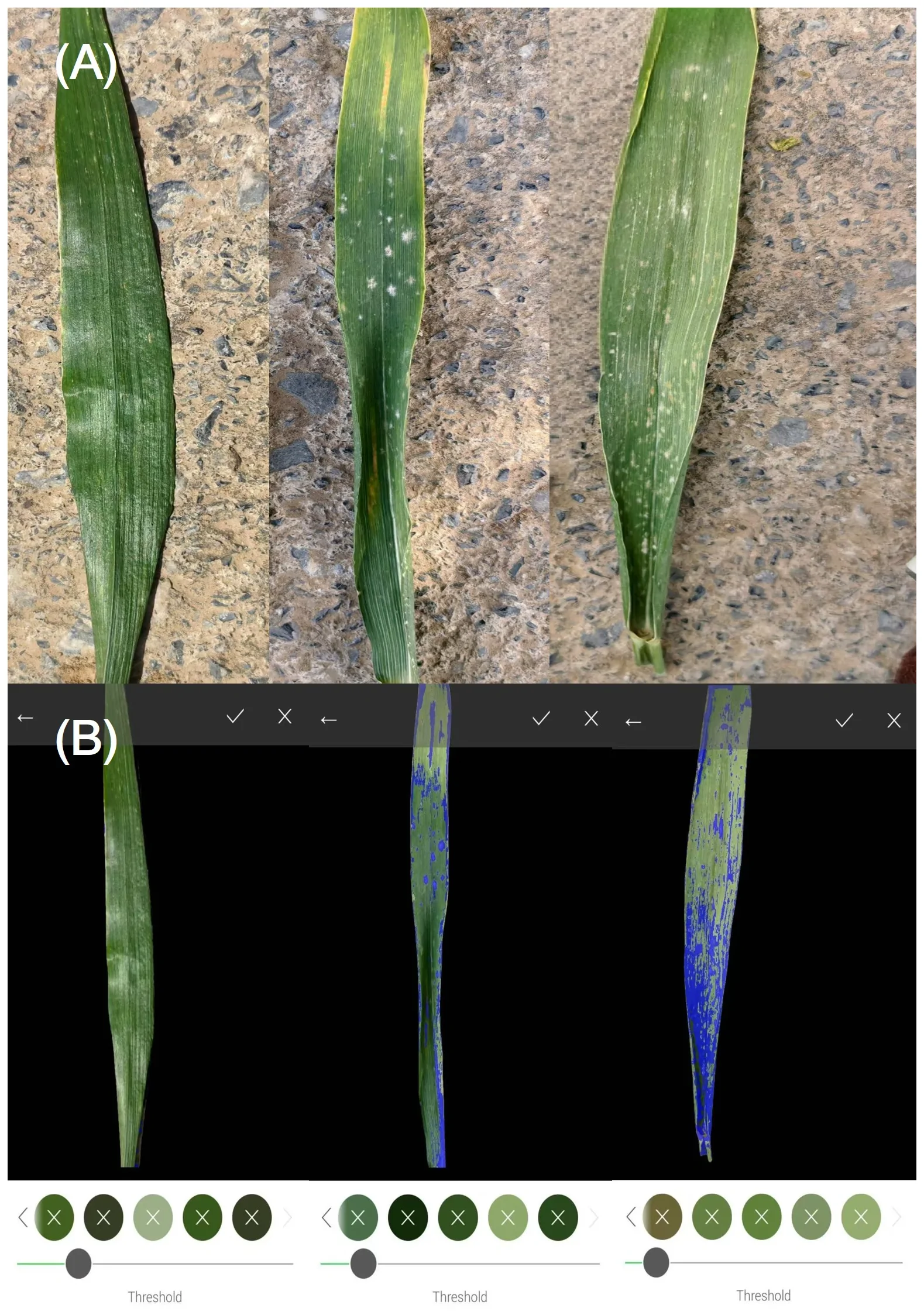
Disease Severity (DS) assessment flowchart. **(A)** Original image captured in the field. **(B)** The pre-processed image entered into the Leaf Doctor software for analysis.

## 3 Model of the LCRNET

### 3.1 The architecture of LCRNet

LCRNet (a convolutional model for large-scale kernel selection based on residual structures) is a deep learning model designed to analyze complex features in wheat chlorophyll fluorescence images. The core of this model is a specially designed Basic Block, which is structurally divided into two parts: a front-end unit for basic feature extraction and a back-end unit for feature enhancement. The front-end unit performs preliminary feature transformation through a sequential stack of standard convolutional layers, batch normalization (BN) layers, and ReLU activation functions. The back-end unit incorporates an LSAF module and a CA module for collaborative feature processing. Through multi-scale contextual awareness and joint spatial-channel weighting, this design enhances the model’s capacity to represent fluorescence features. The overall network architecture is depicted in **Figure 3**. The detailed design of its core feature processing module (the LSAF-CA module) is elaborated in Section 3.3. Compared to traditional convolutional neural networks, the LCRNet model effectively mitigates the vanishing gradient problem in deep networks via the introduced residual connections, thereby improving recognition accuracy in classification tasks.

**Figure 3:**
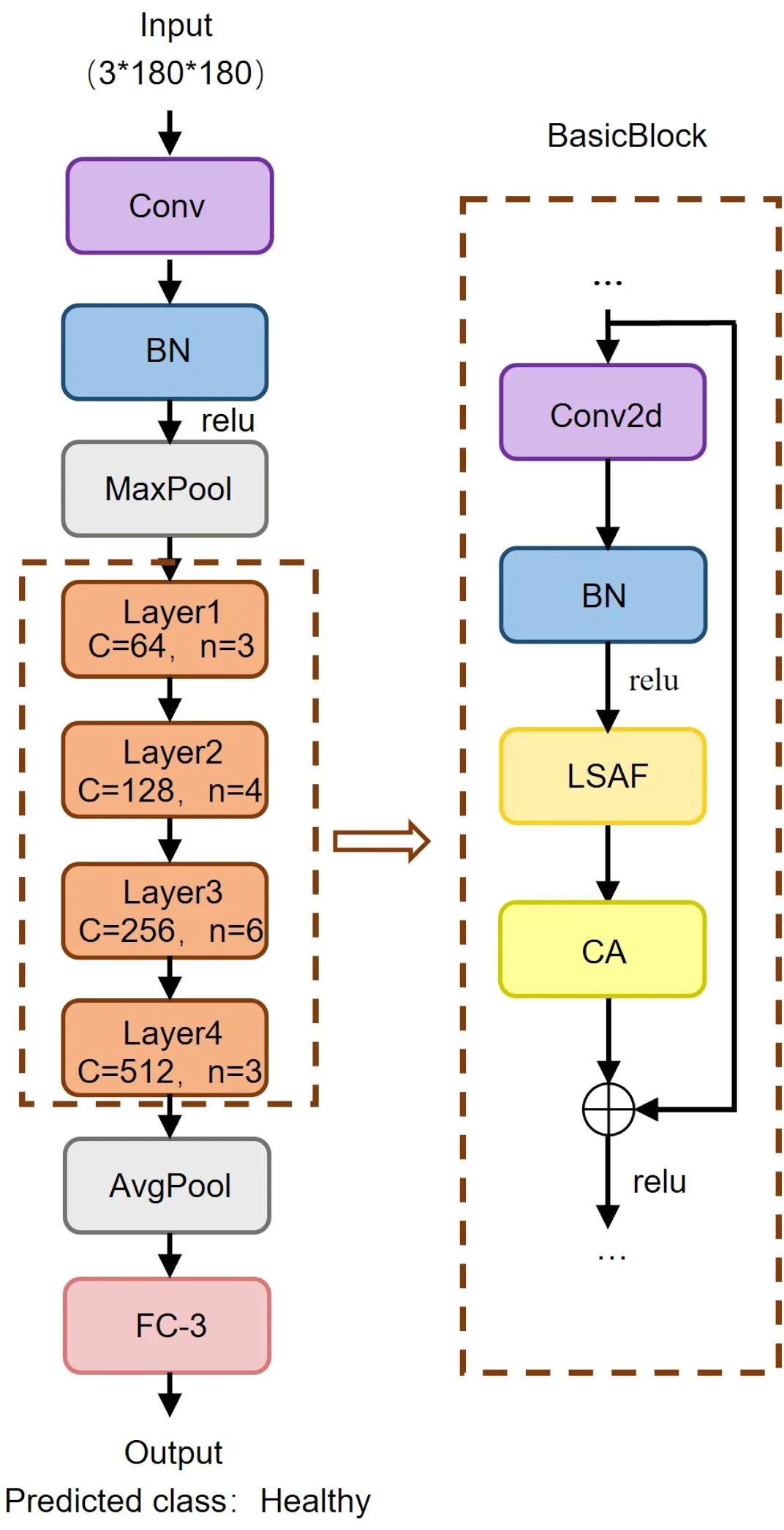
LCRNet model structure diagram. “BN” represents Batch Normalization. “AvgPool” stands for AdaptiveAvgPool2d. ”⊕” represents element-wise addition with identity mapping. The four layers have the same layer structure, all of which are basic blocks. An example path is shown on the right side of the figure.”C” represents depth.”n” represents blocks_num. ”LSAF” represents the LSAF module, and ”CA” represents the CA module.

### 3.2 Layer-by-layer analysis

Based on the architecture design described above, the overall structure of LCRNet comprises an initial feature extraction block, a group of residual blocks, pooling layers, a fully connected layer, and an output layer. This section details the structure and function of each of these components.

Initial Feature Extraction Block: This layer performs preliminary feature extraction and dimensional normalization. The model employs a 7×7 standard convolution kernel (with a stride of 2) to spatially downsample the input three-channel image. Simultaneously, batch normalization (BN) and ReLU activation suppress internal covariate shifts while introducing nonlinearity. This step transforms the input image from 180 × 180 × 3 to a 90 × 90 × 64 feature tensor, laying the foundation for subsequent deep feature learning.

Residual Block Layer Group: This layer conducts deep feature learning via residual connections and attention mechanisms. It comprises four levels (layer1 through layer4), each consisting of multiple Basic Block units. Within each Basic Block, input features first undergo initial feature extraction through a 3×3 convolutional layer, followed by batch normalization and ReLU activation. The features then proceed to the LSAF attention module, which utilizes depthwise separable convolutions with multiple dilation rates for multi-scale feature extraction and adaptive fusion. The fused features are subsequently fed into the CA module. The coordinate-aware mechanism of the CA module enables precise encoding of spatial position information and further feature enhancement.

Pooling Layers: These layers serve the dual purpose of controlling spatial resolution and aggregating features during the extraction process, achieving progressive compression and final unification of feature maps through different pooling strategies. In the early stage, 3×3 max pooling (stride=2) is applied to downsample the 90x90 feature maps generated by the initial block, halving the feature map size while preserving the most salient activations, thereby effectively expanding the receptive field for subsequent layers. At the final stage, adaptive average pooling is used to uniformly compress feature maps of any spatial size down to 1×1. This ensures the fully connected layer receives a fixed-dimension input, facilitating a smooth transition from localized features to a global representation.

Fully Connected Layer: This layer is responsible for mapping the pooled global features to the category space, performing the critical task of converting feature representations into classification decisions. First, the 512×expansion-dimensional feature vector is flattened into a one-dimensional vector. This flattened vector is then passed through the fully connected layer. It performs a linear transformation via a weight matrix, effectively mapping the high-dimensional features onto the category space to generate a num classes-dimensional output vector. This integrates global information across all channels and outputs the classification decision result.

Output Layer: As the model’s final decision stage, this layer transforms the raw logits from the fully connected layer into a normalized probability distribution to yield classification decisions. First, the Softmax function applies exponential normalization, producing a probability distribution that sums to 1. During inference, the index corresponding to the maximum probability is selected as the predicted class, thereby completing the mapping from the feature space to a discrete class label.

### 3.3 Feature processing module design

The LSAF-CA feature processing module, located in the back-end unit of the Basic Block, achieves effective enhancement of chlorophyll fluorescence features through a synergistic mechanism that combines multi-scale receptive field expansion with spatial position awareness. The following subsections detail the technical implementation and merits of each constituent module. To expand the receptive field and enhance multi-scale feature fusion capabilities, this study proposes the Large Selective Adaptive Fusion (LSAF) module. This module captures multi-scale contextual information via multi-branch depthwise separable convolutions and employs a content-based attention mechanism to dynamically weight features from different branches, enabling adaptive receptive field selection. It consists of four key components: a multi-branch depthwise separable convolution, a dynamic weight generator, a feature fusion mechanism, and a feature projector. When a feature map is input to the LSAF module, it is first processed in parallel by three depthwise separable convolution branches for multi-scale feature extraction. Each branch employs an identical 3×3 kernel but with a distinct dilation rate (dilation=1,2,3), corresponding to a different effective receptive field. Each branch’s output is subsequently processed by batch normalization and a ReLU activation function, producing three sets of feature maps enriched with information at different scales. In parallel, the original input features are fed into the dynamic weight generator. Specifically, the input features are first compressed into channel-wise descriptors via global average pooling. These descriptors then pass through a two-layer Multi-Layer Perceptron (MLP), implemented as two successive 1×1 convolutions, for nonlinear transformation. This process outputs a weight vector of dimension (number of branches × number of channels). This vector is reshaped and normalized across the branch dimension using a softmax function, generating a set of attention weights that indicate the importance of each branch for every channel. The outputs from the three convolution branches are then stacked along a new branch dimension. They are multiplied element-wise with their corresponding attention weights and summed, achieving adaptive, content-aware fusion of the multi-scale features. Finally, the fused features pass through a 1×1 convolutional projection layer for channel mixing, followed again by batch normalization and ReLU activation, producing enhanced features with the same dimensionality as the input. This mechanism facilitates optimal receptive field selection and feature enhancement for the input content through dynamic weight allocation. The module structure is illustrated in **Figure 4**.

**Figure 4:**
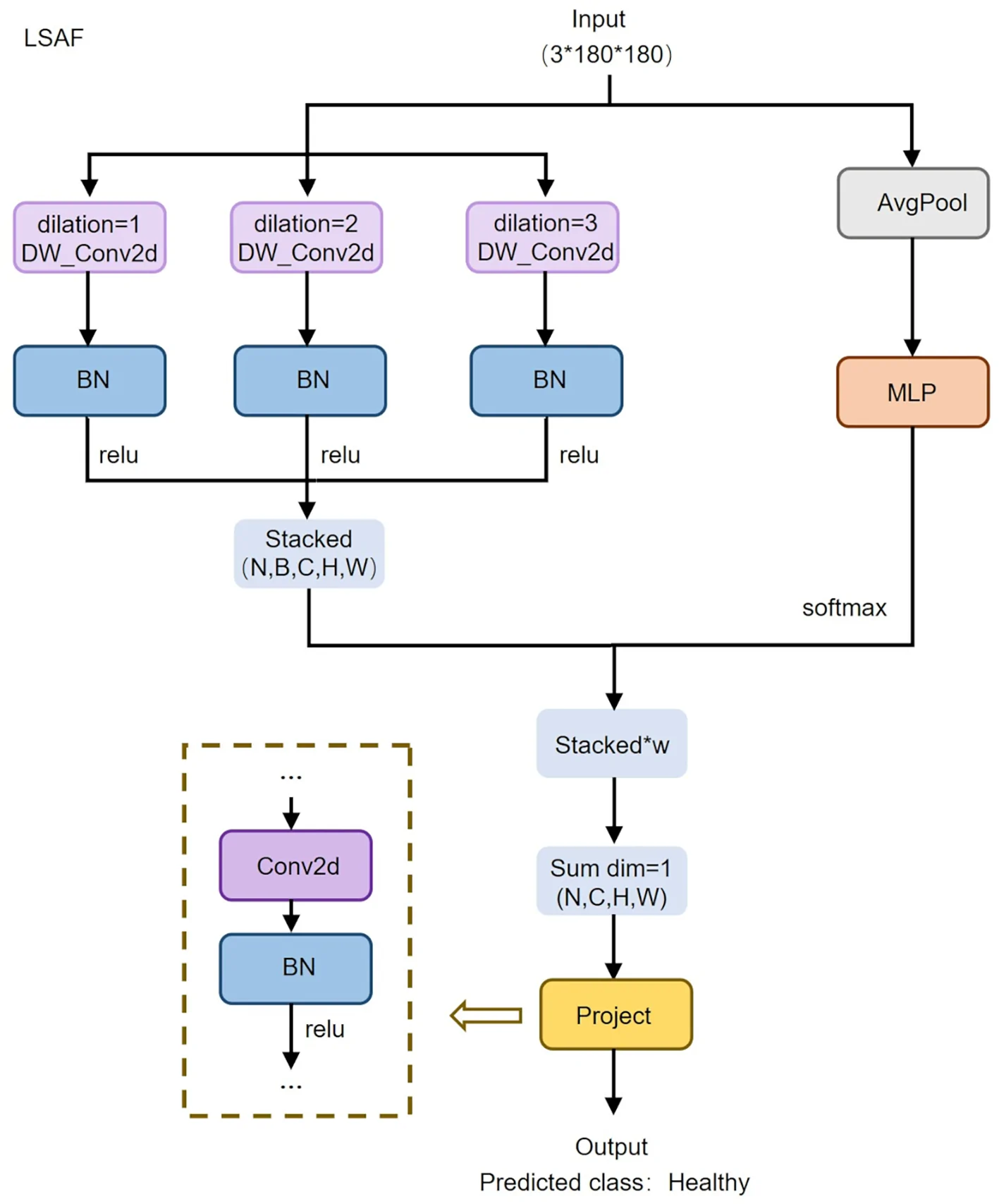
LSAF module flow chart. “Dw_Conv2d” represents Deep Separable Convolution. ”BN” represents Batch Normalization. ”AvgPool” represents AdaptiveAvgPool2d. ”Conv2d” represents 1x1 two-dimensional convolution. ”Stacked” represents stacking operations. ”N” represents Batch_Size. ”C” represents channels. ”H” represents height.”W” represents width.”B” represents branches. ”Sum dim” represents the sum operation on the branch dimension. ”Stacked*w” represents weighted fusion operation. ”Project” represents projection layer, channel mixing and feature transformation for fused features.”MLP” represents attention weight generation network.

To complement the LSAF module, the Coordinate Attention (CA) mechanism is incorporated, which enhances the discriminative power of feature channels via a lightweight, coordinate-aware design. Its core innovation is the explicit modeling of both spatial position information and channel dependencies. This module consists of two main branches: coordinate information embedding and attention generation. The coordinate embedding branch performs adaptive average pooling on the input features separately along the height (H) and width (W) dimensions, generating a pair of direction-aware feature encodings. These encoded features are then concatenated and passed through a 1×1 convolutional layer, followed by batch normalization and ReLU activation to generate an intermediate feature representation that integrates directional spatial information. Finally, they are decomposed into independent spatial encoding vectors along the width and height dimensions. This process enables the module to capture joint channel-spatial dependencies within feature maps. The attention generation branch takes the spatial encoding vectors as input. They are processed through another 1×1 convolution and a Sigmoid activation function to generate a channel attention weight map. This weight map is then applied to the original input features via element-wise multiplication, selectively enhancing the most informative channels. The module structure is illustrated in **Figure 5**.

**Figure 5:**
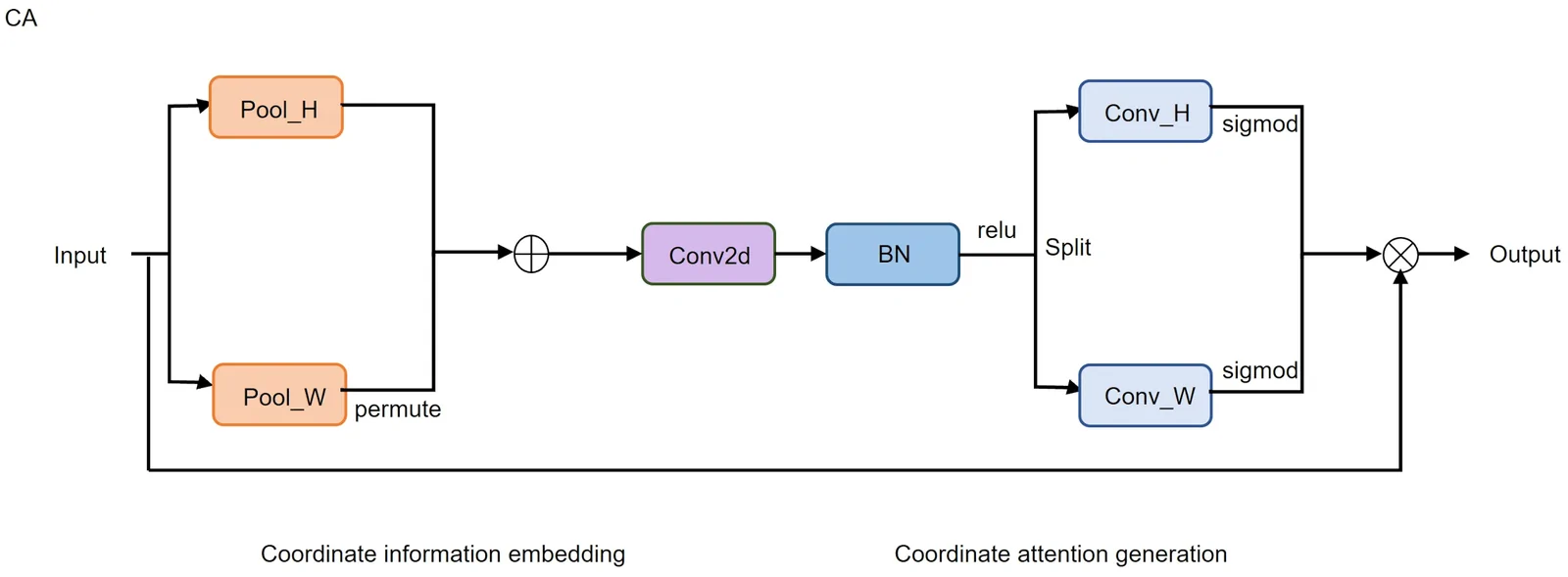
CA module flow chart. “Pool_W” represents adaptive average pooling along the height, outputting (N, C, 1, W). “Pool_H” represents adaptive average pooling along the width, outputting (N, C, H, 1). “Permute” represents the transposed output dimension of Pool_W, making it easy to concatenate with Pool_H. “Conv_W” represents a 1x1 convolution that generates attention weights in the width direction and outputs (N, C, 1, W). “Conv_H” represents a 1x1 convolution that generates attention weights in the height direction and outputs (N, C, H, 1). “⊕” indicates concat along the height dimension, integrating the two-way pooling feature. “⊗” indicates the weighting operation of the matrix. “BN” represents Batch Normalization.

In summary, this study establishes a collaborative feature processing mechanism grounded in multi-scale feature extraction and channel-discriminative enhancement. The LSAF module expands the effective receptive field through its multi-branch, adaptive fusion design, enabling it to adaptively capture multi-scale contextual information to provide the CA module with multi-scale spatially refined features. The CA module, in turn, performs position-sensitive channel recalibration on these features through its explicit height- and width-wise coordinate encoding. This introduces directional awareness and suppresses redundant channels. Through this synergistic “multi-scale refinement followed by channel-discriminative enhancement” workflow, the two modules collectively contribute to an effective model adept at analyzing chlorophyll fluorescence images against complex backgrounds. This integrated approach enhances the model’s adaptability and discriminative capability in complex scenarios while maintaining parameter efficiency.

## 4 Results

### 4.1 Data preprocessing section

To reveal the interrelationships among chlorophyll fluorescence parameters and disease severity, Pearson correlation analysis was performed on the fluorescence data from 94 samples, with the results presented as a heatmap (**Figure 6**; all *p* -values *<* 0.001). The analysis revealed significant correlations among the key fluorescence parameters. QYmax showed a moderate positive correlation with Rfd (r = 0.50) and a moderate negative correlation with Y(NO) (r=-0.43). This result confirmed the trade-off competition between photochemical energy conversion and non-regulatory energy dissipation pathways under disease stress, a phenomenon that can be effectively characterized using the complementary quantum yield framework proposed by Klughammer and Schreiber (2008). Y(NO) showed a significant negative correlation with Rfd (r =-0.83), indicating that PSII function rapidly declined or even reached irreversible damage under severe powdery mildew infection. The heatmap (**Figure 6**) further revealed the strength of the correlations between each fluorescence parameter and disease severity. QYmax exhibited the strongest negative correlation with disease severity, followed by Rfd (r=-0.77; -0.38, respectively), while Y(NO) showed a moderate positive correlation with disease severity (DS) (r =0.40). In summary, QYmax was highly coupled with disease severity and represented the most promising diagnostic indicator.

**Figure 6:**
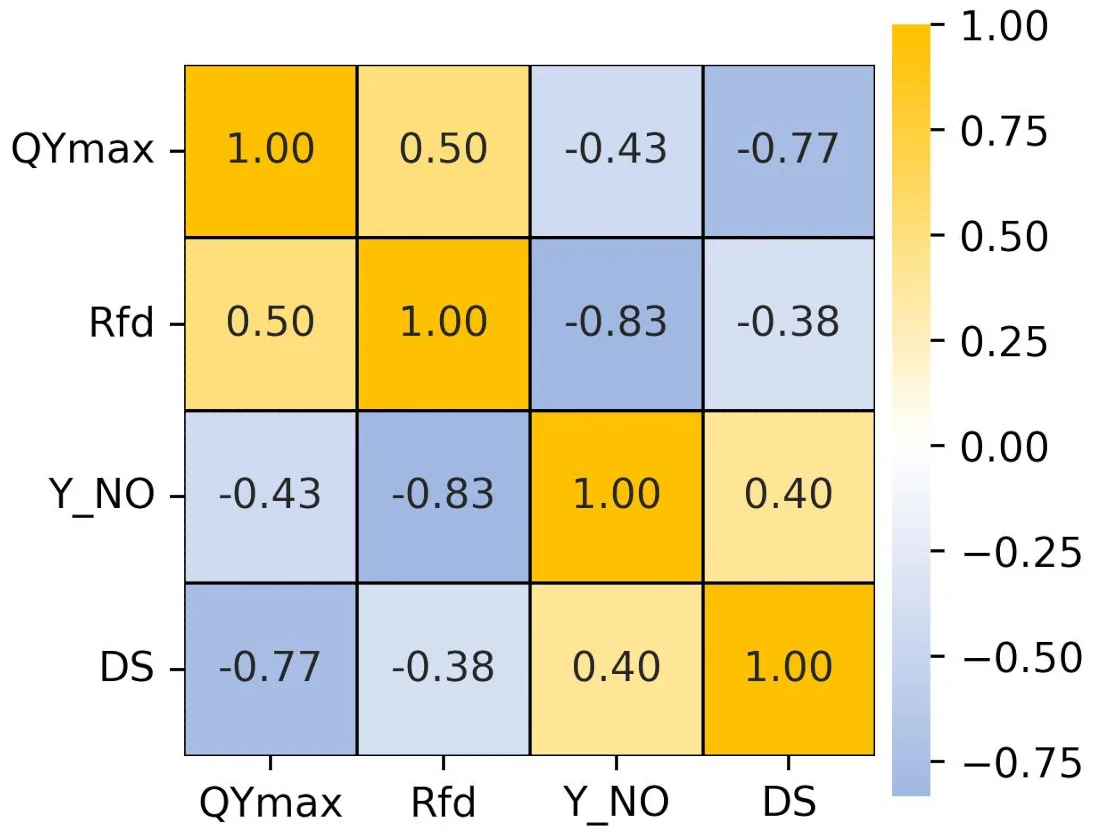
Thermodynamic diagram of correlation between fluorescence indexes and disease degree.

However, because two samples were missing from Plot 6, this plot no longer had a complete set of four leaf measurements for calculating a reliable average. Therefore, Plot 6 was excluded entirely from the subsequent comparative analysis of disease progression across plots. To investigate the dynamic performance of various fluorescence parameters during disease progression, the temporal variations of QYmax, Rfd, and Y(NO) at key wheat growth stages were analyzed. **Figure 7** illustrated the temporal trends of these parameters across the seven plots at three key wheat growth stages (the heading, flowering, and grain-filling stages of wheat). As shown in **Figure 7A**, QYmax exhibited a consistent and significant downward trend in nearly all plots throughout the observation period. Its value continuously decreased with disease progression, visually reflecting the intensifying inhibition of photosynthesis and the decline in overall photosynthetic efficiency. Although the downward trend was consistent, the rate of decline and the final level of QYmax varied among plots. Overall, the maximum decrease in QYmax was approximately 7.0. Among the plots, Plots 5 and 7 showed the most pronounced changes: the former displayed the fastest decline between the heading stage and the flowering stage, while the latter experienced the steepest drop between the flowering stage and the grain-filling stage. The remaining plots demonstrated relatively smaller changes with more gradual trends. In summary, QYmax demonstrated high sensitivity and consistency in response throughout disease progression. Rfd also exhibited a declining trend with disease advancement (**Figure 7B**), with a more pronounced decrease observed between the heading and flowering stages compared to the subsequent transition to the grain-filling stage. This indicated that as the disease progressed, the repair capacity of PSII might be insufficient to compensate for the damage. Compared to QYmax, the response of Rfd exhibited greater variability and spatial heterogeneity across different plots. For instance, plots 1, 3, and 5 did not exhibit the consistent, significant decline observed for QYmax. Thus, while Rfd could reflect stress at a local level, its overall response lacked consistency, limiting its role as a robust indicator for disease assessment. Y(NO) showed an overall upward trend with disease progression (**Figure 7C**). Except for specific fluctuations in plots 1, 2, and 3, most other plots exhibited insignificant changes across the different periods. Overall, Y(NO) reflected non-regulated energy dissipation under severe infection but lacked sufficient stability. Among all observed plots, Plot 1 exhibited distinctly different response patterns. Its QYmax and Y(NO) first decreased and then increased, while its Rfd continuously rose, contradicting the overall downward trend. A comparative analysis of the dynamic trends of the three parameters revealed that QYmax exhibited the highest consistency, clear directionality, and good discriminatory power in its temporal variation. Notably, Plot1 showed response parameters that were markedly divergent from the overall trend.

**Figure 7:**
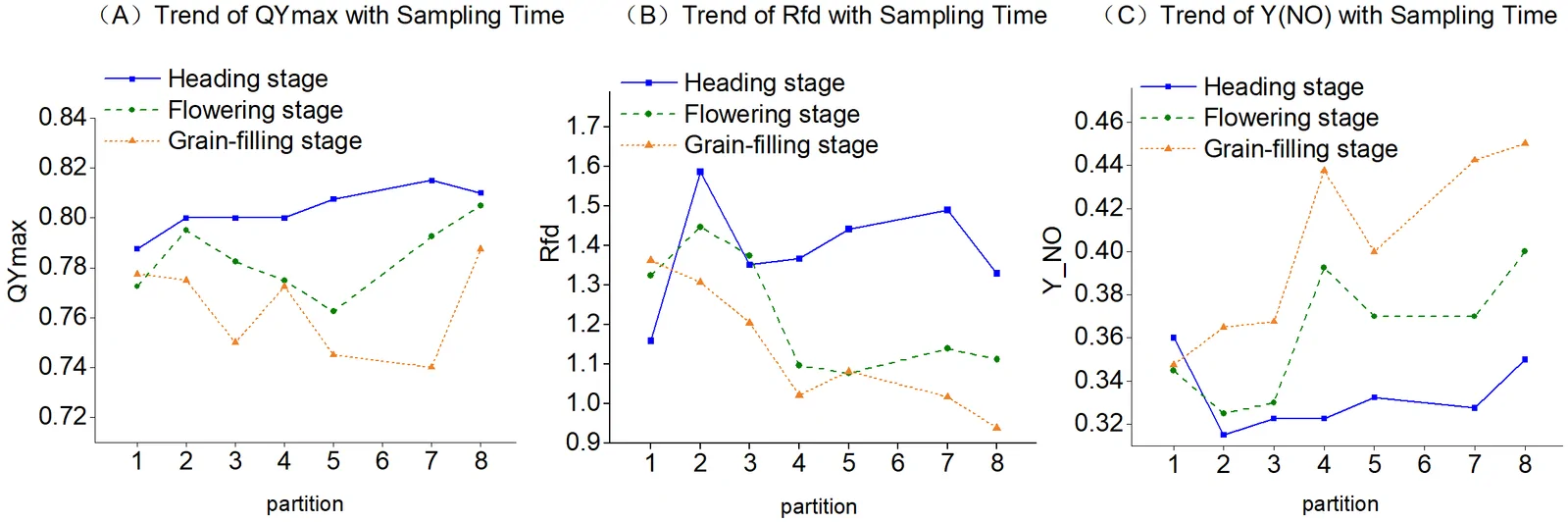
Trend chart of fluorescence indicators over sampling time. The horizontal axis represents plots, the vertical axis represents the values of each fluorescence indicator, and the different colors of the lines represent different time points. The three figures represent, in sequence: **(A)** QYmax. **(B)** Rfd. **(C)** Y_NO.

To statistically quantify the ability of each indicator to discriminate between healthy and diseased states (**Figure 8**), grouped box plots were constructed. In this binary physiological comparison, samples with disease severity ≥ 5% (i.e., the mild and moderate classes) were pooled as the “diseased” group. This analysis further supported the conclusions from the dynamic trend analysis. As shown in **Figure 8A**, the median QYmax exhibited a declining trend over time, with a more pronounced decrease in the diseased samples. Its box showed no overlap between the healthy and diseased groups, demonstrating excellent discriminatory power. In contrast, while Rfd also exhibited a downward trend, its boxes for the healthy and diseased groups showed significant overlap (**Figure 8B**), indicating a weaker discriminatory ability. Y(NO) showed only an upward trend over time, with more significant overlap between the boxes of the healthy and diseased groups (**Figure 8C**), indicating a limited ability to distinguish between the two states. The box-and-whisker plot analysis further confirmed that QYmax was the optimal fluorescence indicator for differentiating between healthy and diseased wheat leaves. It should be noted that this binary comparison represents a preliminary physiological analysis; the subsequent classification model addresses a three-class problem (healthy, mild, and moderate) to provide finer-grained severity assessment.

**Figure 8:**
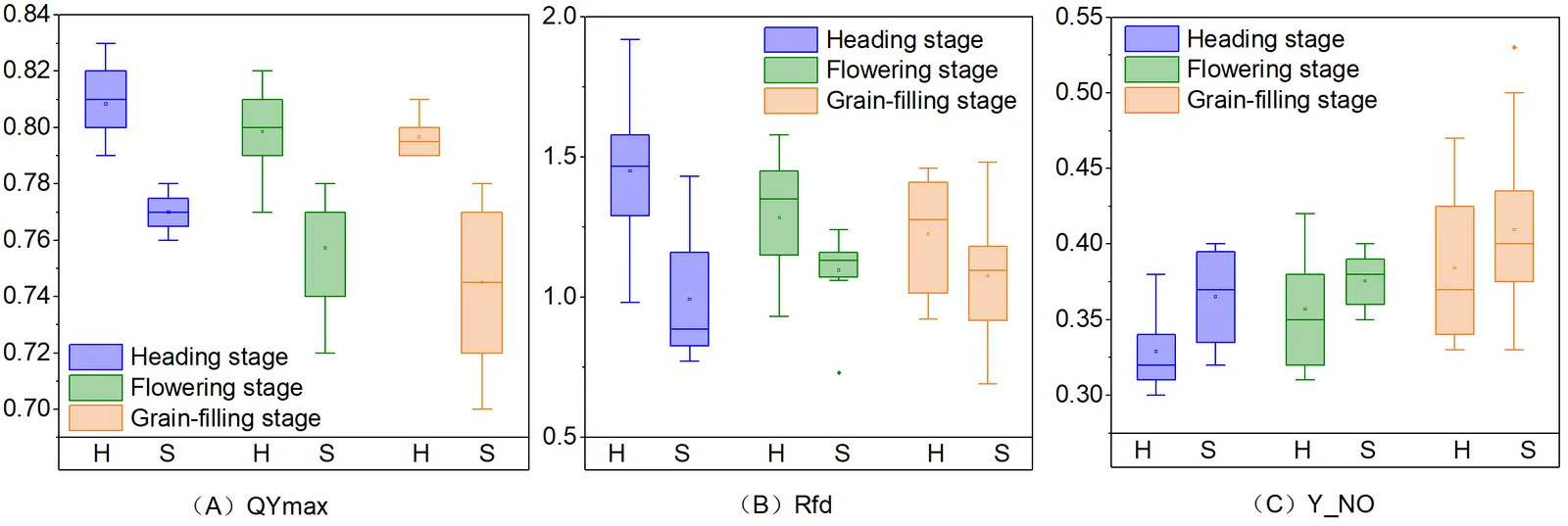
Boxplots of fluorescence indicators for healthy and diseased populations at different sampling time points. Population at different sampling time points. In this binary physiological comparison, samples with disease severity ≥ 5% (i.e., mild and moderate) are merged into the “diseased” group, while samples with lesion coverage less than 5% are classified as the “healthy” group. These three numbers represent: **(A)** QYmax. **(B)** Rfd. **(C)** Y_NO.

In summary, throughout disease progression, QYmax demonstrated the highest consistency in response and temporal sensitivity. From a temporal perspective, it exhibited a clear unidirectional decline pattern across the entire observation period, consistently indicating a worsening disease trend. Regarding state differentiation, the numerical distributions of healthy and infected samples showed high dispersion and minimal overlap, revealing exceptional classification potential. While the above analysis employed a binary comparison (healthy vs. diseased), the three-class classification framework (*healthy*, *mild*, and *moderate*) adopted in the subsequent modeling further enables fine-grained discrimination among disease severity levels.

Based on the results of the above analysis, we selected the fluorescence images corresponding to QYmax—the metric most strongly correlated with disease severity—to construct a fluorescence dataset for wheat powdery mildew. In accordance with the classification guidelines and taking into account the distribution of experimental data and modeling requirements, we consolidated and simplified the classification criteria into three levels: healthy, mild, and moderate (Li et al., 2025). The specific classification criteria were as follows: lesion coverage of 5–30% was classified as mild disease; lesion coverage of *>* 30% was classified as moderate disease. Representative sample images for each category were shown in **Figure 9**. When constructing the dataset, we strictly adhered to the principles of sample-level partitioning. To ensure that the class distributions in the training, validation, and test sets remained consistent with those in the original dataset, this study adopted a hierarchical partitioning strategy. First, the 94 individual leaf images were partitioned into an 8:1:1 ratio for each class; subsequently, the subsets were merged to construct the overall training, validation, and test sets. Only after this partitioning was completed did we perform local tile extraction (180 ×180px), based on different regions and data augmentation operations on individual leaf images within the sets. The specific data augmentation methods used are shown in **Figure 10**. After cropping, the number of samples in each class was as follows: 310 for the “healthy” class, 207 for “mild,” and 126 for “moderate.” A total of 3,181 samples were ultimately obtained for model input. Care was taken to ensure that data from the same leaf never spanned different sets. To ensure the validity of the improved models, all recognition models utilized the same dataset, which was partitioned in the same manner, thereby completing the construction of the wheat powdery mildew fluorescence image dataset.

**Figure 9:**
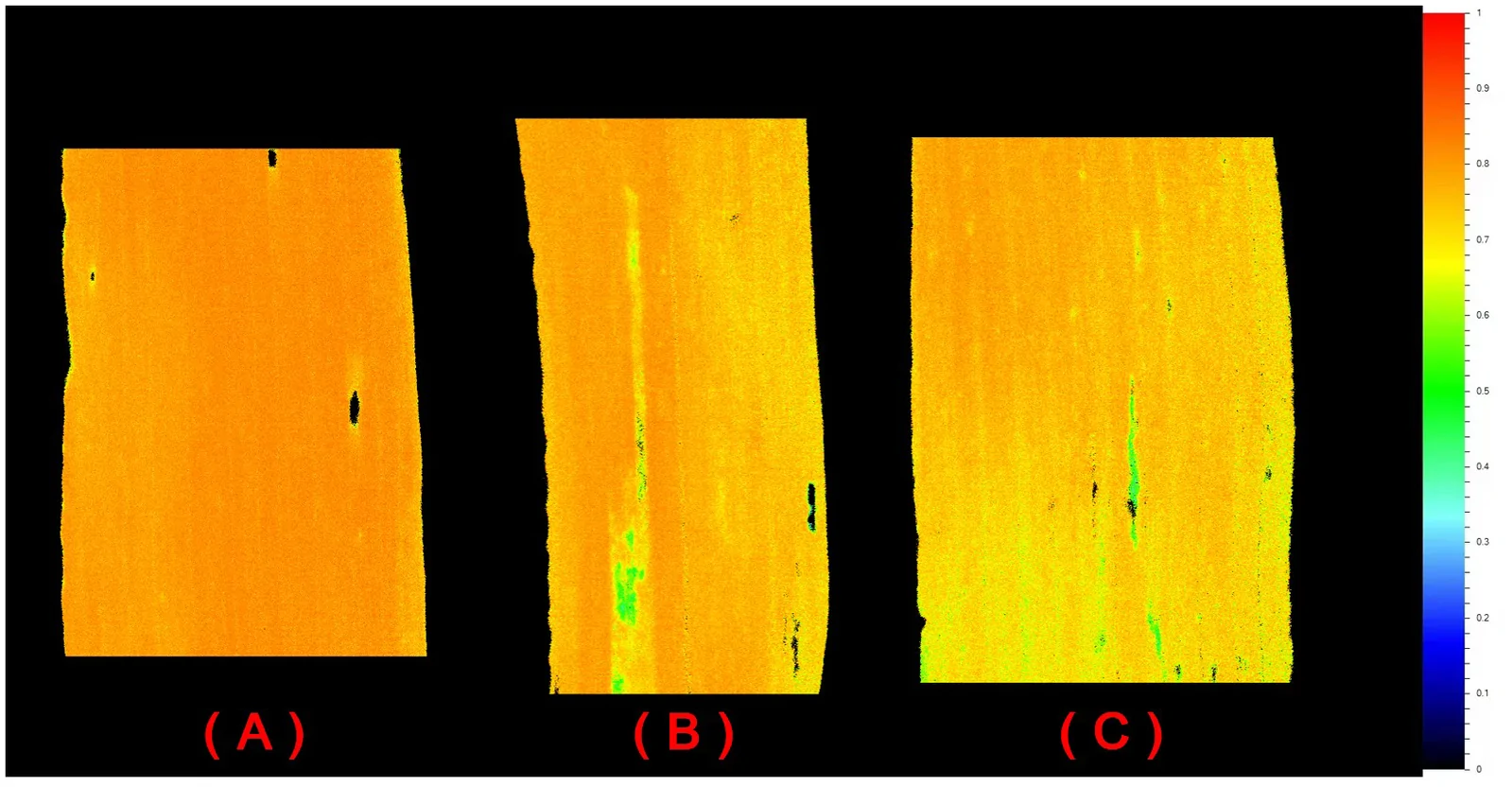
Fluorescent schematic diagram classified by disease severity. The three figures represent, in sequence: **(A)** Healthy. **(B)** Mild disease. **(C)** Moderate disease.

**Figure 10:**
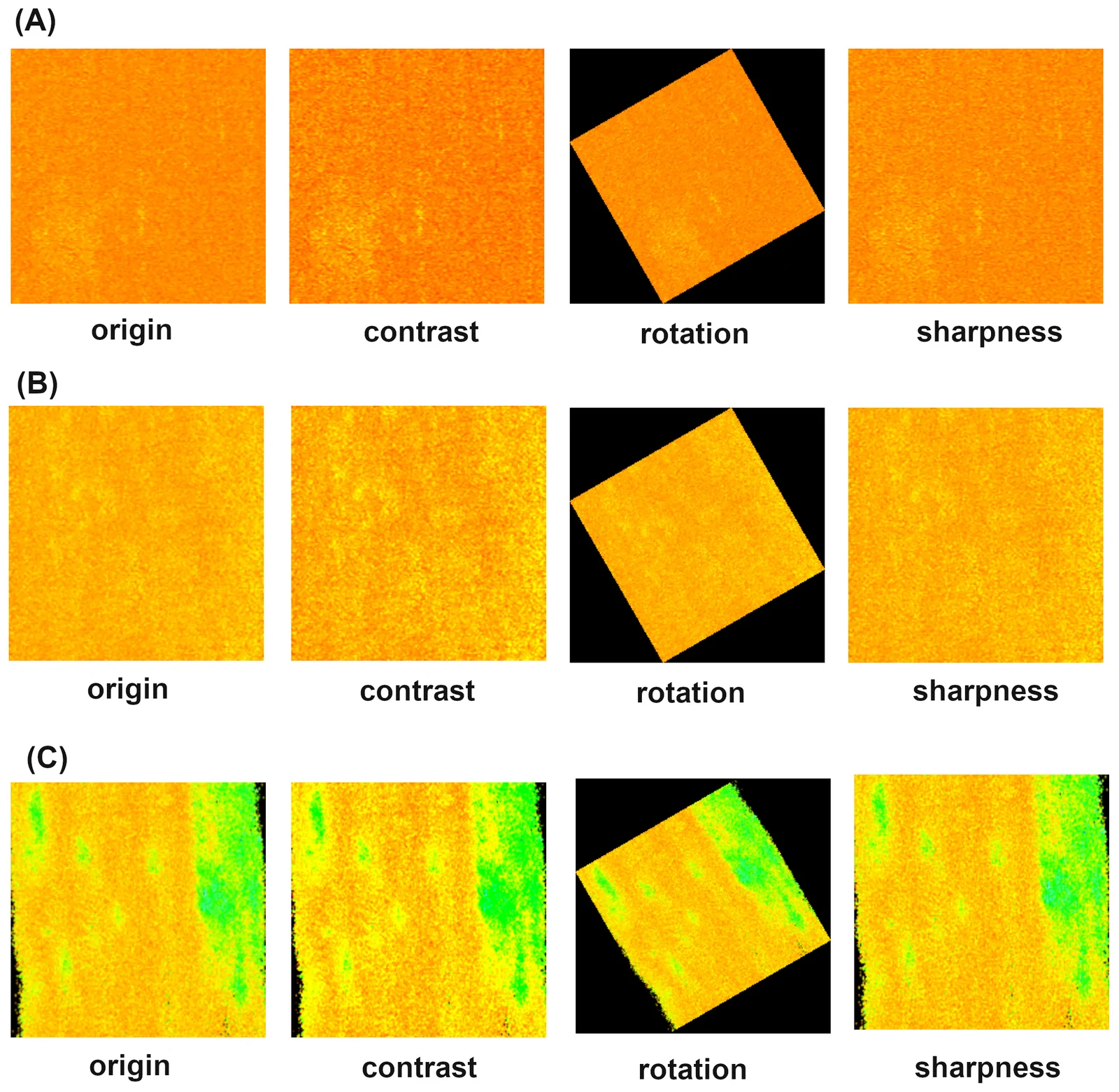
Data augmentation strategies. **(A)** Healthy. **(B)** Mild disease. **(C)** Moderate disease.

### 4.2 Parameter settings

The experiments were conducted using Python 3.9 and CUDA 11.8 within PyCharm 2023.2. The hardware configuration included a 13th Gen Intel Core i7-13650HX CPU and an NVIDIA GeForce RTX 4060 GPU (6 GB). Throughout the research, the PyTorch framework and Torchvision library were extensively utilized, integrated with various image processing tools to enable real-time model construction, modification, and debugging. This capability significantly enhanced the efficiency of model development and optimization, providing strong support for building an effective wheat powdery mildew classification system. The parameter settings for this study were shown in **Table 1**.

**Table 1:** Parameter setting.

Parameter name	Value	Parameter name	Value
Data size	180 × 180	Optimizer	Adam
Epoch	120	Loss Function	CrossEntropyLoss
Learning Rate	0.0001	Feature Dimension	512
Batch size	32	Conv Kernel	7 × 7,3 × 3,1 × 1

### 4.3 Data balidity analysis

To fully exploit the limited dataset for both training and validation, this study employed 5-fold cross-validation, with the quantitative results summarized in **Table 2**. To further investigate the model’s decision-making mechanism and ensure interpretability, we applied the Gradient-weighted Class Activation Mapping (Grad-CAM) technique (Panwar et al., 2020). By directing task-specific gradients into the final convolutional layer, Grad-CAM generated a coarse localization map that visually highlighted the critical regions contributing to the prediction. As shown in **Figure 11**, these heatmaps provided intuitive insights into the feature extraction process, demonstrating the model’s ability to focus on subtle yet discriminative details in the image features. Such alignment between the model’s attention and domain knowledge further attests to the quality and informativeness of our dataset.

**Table 2:** Performance results of the 5-fold cross-validation.

Fold	Accuracy (%)	Precision (%)	Recall (%)	F1 (%)
Fold 1	94.7	94.5	93.2	93.8
Fold 2	91.3	90.3	91.0	90.6
Fold 3	92.1	92.5	92.5	92.3
Fold 4	91.7	91.7	91.7	91.6
Fold 5	93.6	92.1	93.8	92.8
**Mean±Std**	**92.7 ± 1 .26**	**92.2 ± 1 .37**	**92.4 ± 1 .02**	**92.3 ± 1 .08**

Bold values indicate the mean and standard deviation of the results.

**Figure 11:**
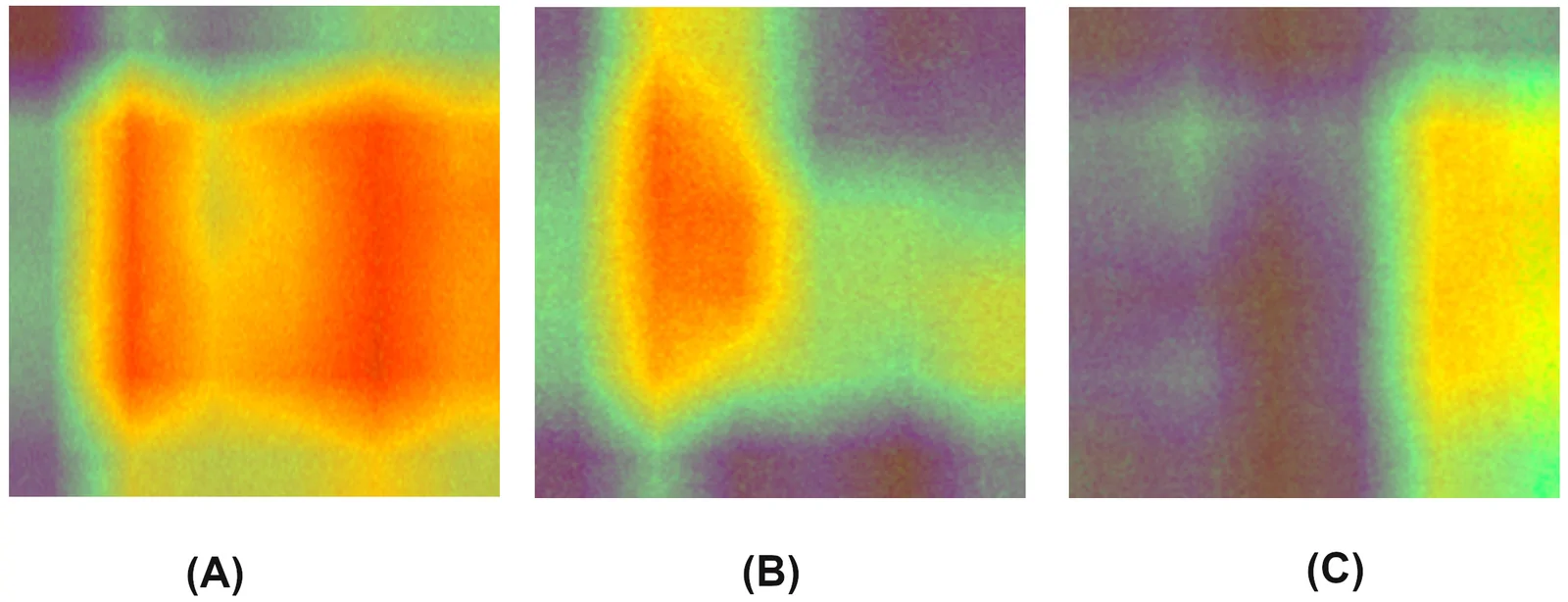
Visual heat map of feature attention. **(A)** Healthy. **(B)** Mild disease. **(C)** Moderate disease.

### 4.4 Evaluation indicators

To systematically evaluate the performance of the LCRNet model in wheat powdery mildew classification, this study employed a comprehensive multidimensional metric system comprising accuracy, precision, recall, F1-score, and Cross-Entropy loss.True positives (TP) denoted the number of samples correctly identified as belonging to a given class. In the three-class classification framework (healthy, mild, and moderate), metrics were computed for each class using a one-vs-rest strategy and then macro-averaged to obtain overall scores. Accuracy measured the proportion of correct predictions made by the model overall, serving as one of the most intuitive and commonly used performance metrics (Equation 1).

(1) $$\text{Accuracy}=\frac{TP+TN}{TP+TN+FP+FN}.$$

False Negatives (FN) denoted the number of samples that were actually from a given class but misclassified as another class; False Positives (FP) represented samples from other classes incorrectly labeled as the given class; True Negatives (TN) indicated the number of samples from other classes correctly classified as not belonging to the given class. Cross-entropy loss effectively measured the discrepancy between the predicted probability distributions and actual distributions. This loss function was inherently suited for multi-class classification tasks. Its convergence dynamics during training were visualized through loss value change curves in the experimental section.

Precision indicated the proportion of true positives among samples predicted as positive by the model. A higher precision indicated a lower proportion of false positives, signifying more reliable predictions (Equation 2).

(2) $$\text{Precision}=\frac{TP}{TP+FP}.$$

Recall measured a model’s ability to identify all true positive samples, reflecting its coverage of the target class and sensitivity to key categories (Equation 3).

(3) $$\text{Recall}=\frac{TP}{TP+FN}.$$

The F1-score was the harmonic mean of precision and recall, designed to balance the trade-off between these two metrics. In scenarios with class imbalance, the F1-score avoided the limitations of relying solely on accuracy as a single evaluation metric (Equation 4).

(4) $$\text{F}1-\text{score}=\frac{2\cdot \text{Precision}\cdot \text{Recall}}{\text{Precision}+\text{Recall}}.$$

### 4.5 Ablation study

The LCRNet model integrated residual connections, LSAF modules, and CA modules to synergistically optimize feature extraction and enhancement performance. To evaluate the importance of each core component of LCRNet, we designed and conducted ablation experiments. The experimental results further validated the effectiveness of the LCRNet model. Under identical training conditions, we sequentially incorporated the enhancement modules and used various metrics to demonstrate performance at different stages of improvement. The results of the ablation experiments were shown in **Figure 12**.

**Figure 12:**
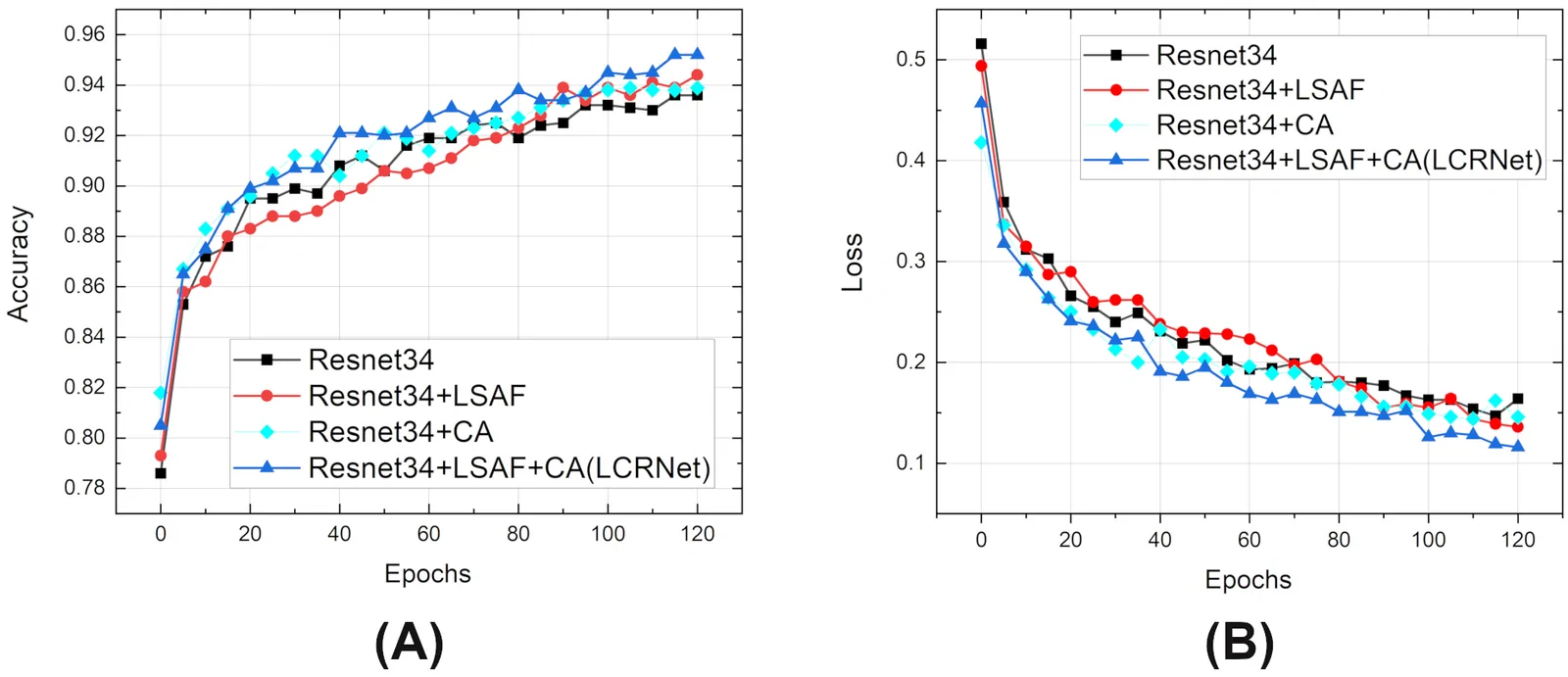
Ablation study on the proposed model: Training accuracy and loss curves. **(A)** Accuracy. **(B)** Loss value.

Experimental results showed that, after introducing the LSAF module, the model—by expanding its receptive field—was able to adaptively capture multiscale contextual information related to plant diseases, thereby enhancing its multiscale feature fusion capabilities. The loss value increased slightly, but the overall accuracy rose to 93.7%. By integrating these two types of attention mechanisms, the final model achieved an accuracy of 94% and reduced the loss to 0.245. Detailed ablation experiment results were summarized in **Table 3**.

**Table 3:** Ablation experiment results.

Method	Loss	Accuracy (%)	Precision (%)	Recall (%)	F1 (%)
ResNet34	0.248	91.2	91.4	91.1	91.2
ResNet34 + LSAF	0.264	93.7	93.7	93.7	93.7
ResNet34 + CA	0.385	90.6	91.6	90.6	90.7
**ResNet34 + CA + LSAF (LCRNet)**	**0.245**	**94.0**	**94.4**	**94.0**	**94.1**

Bold values indicate the effectiveness of the proposed model using design modules.

The baseline network, which lacked any attention modules, achieved a precision of 91.2% and a loss of 0.248 on the validation set; its fixed convolutional kernels limited the network’s effective receptive field, resulting in the model’s inability to adequately capture lesion context across scales and over large spatial spans. To evaluate the individual contribution of the attention mechanisms, the Coordinate Attention (CA) module was first integrated into the baseline network, yielding an accuracy of 90.6% and a loss of 0.385. After introducing the LSAF module, the model’s accuracy and F1 score improved by 2.5%, with precision increasing by 2.3% and recall by 2.6%. With the further introduction of the Coordinate Attention (CA) module, the loss value decreased from 0.264 to 0.245, achieving an F1 score of 94.1% and a precision of 94.4%, indicating that the CA module worked in concert with the LSAF module to effectively reduce the 405 model’s errors in multiscale lesion identification and localization.

### 4.6 Comparative experiment

To systematically evaluate the performance differences between the LCRNet model and other mainstream models, comparative experiments were conducted using the same dataset of fluorescent images of wheat powdery mildew. The selected comparison models encompassed representative convolutional neural network models, including AlexNet (Krizhevsky et al., 2012), GoogLeNet (Szegedy et al., 2015), EfficientNet-B0 (Tan and Le, 2019), EfficientNetV2 (Tan and Le, 2021), and ConvNeXt (Liu et al., 2022).

AlexNet represented a typical model of early deep convolutional networks, extracting features through large convolutional kernels in the first layer and fully connected structures; GoogLeNet achieved multiscale parallel processing through Inception modules; the EfficientNet series balanced scale and performance through a composite scaling strategy; and ConvNeXt demonstrated new potential by integrating convolutional and transformer concepts. Although the aforementioned comparison models each had their own advantages within their respective design paradigms, LCRNet achieved a performance breakthrough in this task through the synergistic mechanism of its CA and LSAF modules. As shown in **Figure 13**, LCRNet achieved an F1 score of 94.1%. At the same time, its parameter count was kept to 12.11M, maintaining good computational efficiency. This indicated that the LCRNet model adapted better to the spatial heterogeneity and channel feature correlations in fluorescence images than general-purpose models. The LCRNet model had a moderate computational complexity (GFLOPs); although its response speed was slower, this trade-off was worthwhile given the performance gains it delivered.

**Figure 13:**
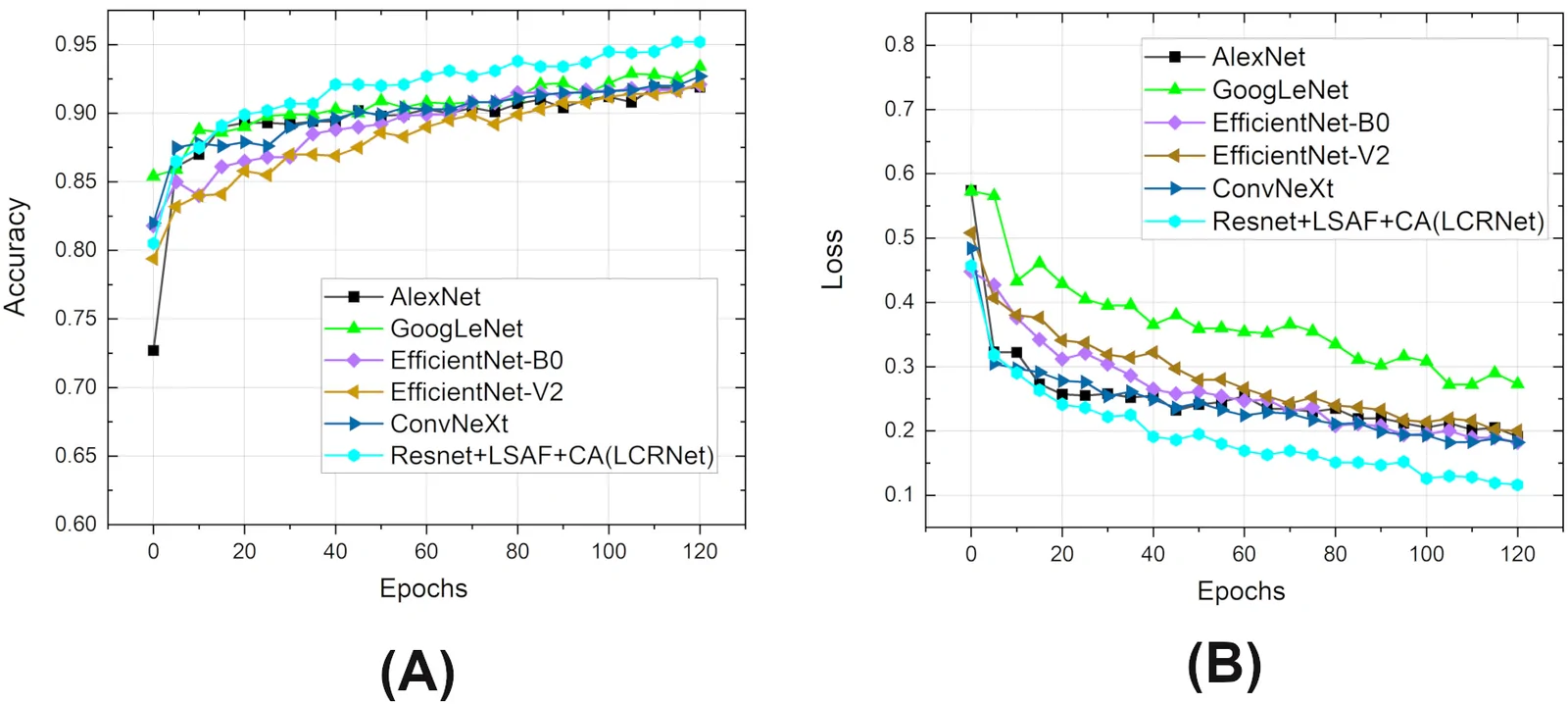
Comparison of training accuracy and loss curves among different models. **(A)** Accuracy. **(B)** Loss value.

As shown in **Table 4**, compared with other models, the LCRNet model performed well in terms of performance-to-parameter ratio, achieving an F1 score of 94.1%, which was 3.2 percentage points higher than that of the EfficientNetV2 model. In terms of parameter efficiency, although the LCRNet model had a higher number of parameters (12.11M) than the EfficientNet-B0 model (4.01M), the GoogLeNet model (5.97M), and the AlexNet model (9.34M), this computational overhead was still only 60.0% and 43.5% of that of the EfficientNetV2 and ConvNeXt models, respectively. Therefore, the LCRNet model struck a good balance between improving prediction performance and avoiding significant computational overhead.

**Table 4:** Comparison of experimental results.

Method	Loss	Accuracy (%)	Precision (%)	Recall (%)	F1 (%)	Params (M)	GFLOPs (G)	Latency (ms)
AlexNet	0.330	91.5	92.9	91.5	91.4	9.34	0.19	0.49
GoogLeNet	0.311	93.1	93.2	93.1	93.1	5.97	0.98	4.03
EfficientNet-B0	0.388	90.9	93.1	90.8	90.8	4.01	0.29	5.99
EfficientNetV2	0.282	91.2	92.7	91.1	90.9	20.18	2.00	11.78
ConvNeXt	0.269	91.5	92.8	91.5	91.6	27.80	2.69	4.08
**LCRNet**	**0.245**	**94.0**	**94.4**	**94.0**	**94.1**	**12.11**	**1.46**	**15.16**

Bold values indicate the best performance among all compared methods.

In summary, LCRNet performed well in terms of accuracy, parameter efficiency, and overall performance; minor differences from other models on specific evaluation metrics did not significantly diminish its overall effectiveness in detecting wheat powdery mildew. The confusion matrix below, generated from the disease classification task, illustrated LCRNet’s accuracy and challenges in correctly identifying individual diseases, and compared LCRNet with AlexNet, GoogLeNet, EfficientNet-B0, EfficientNetV2, and ConvNeXt.

As shown in **Figure 14**, the confusion matrix summarized the classification results of each model. The rows represented the predicted classes, and the columns represented the ground-truth classes. The diagonal elements represented the probability of correctly identifying a disease category, while the off-diagonal elements reflected the probability of misclassification. The confusion matrix indicated that, although LCRNet’s accuracy for individual categories was lower than that of other models, it maintained excellent and balanced performance across all categories, demonstrating more robust overall recognition performance. This observation supported the effectiveness of LCRNet in wheat powdery mildew recognition.

**Figure 14:**
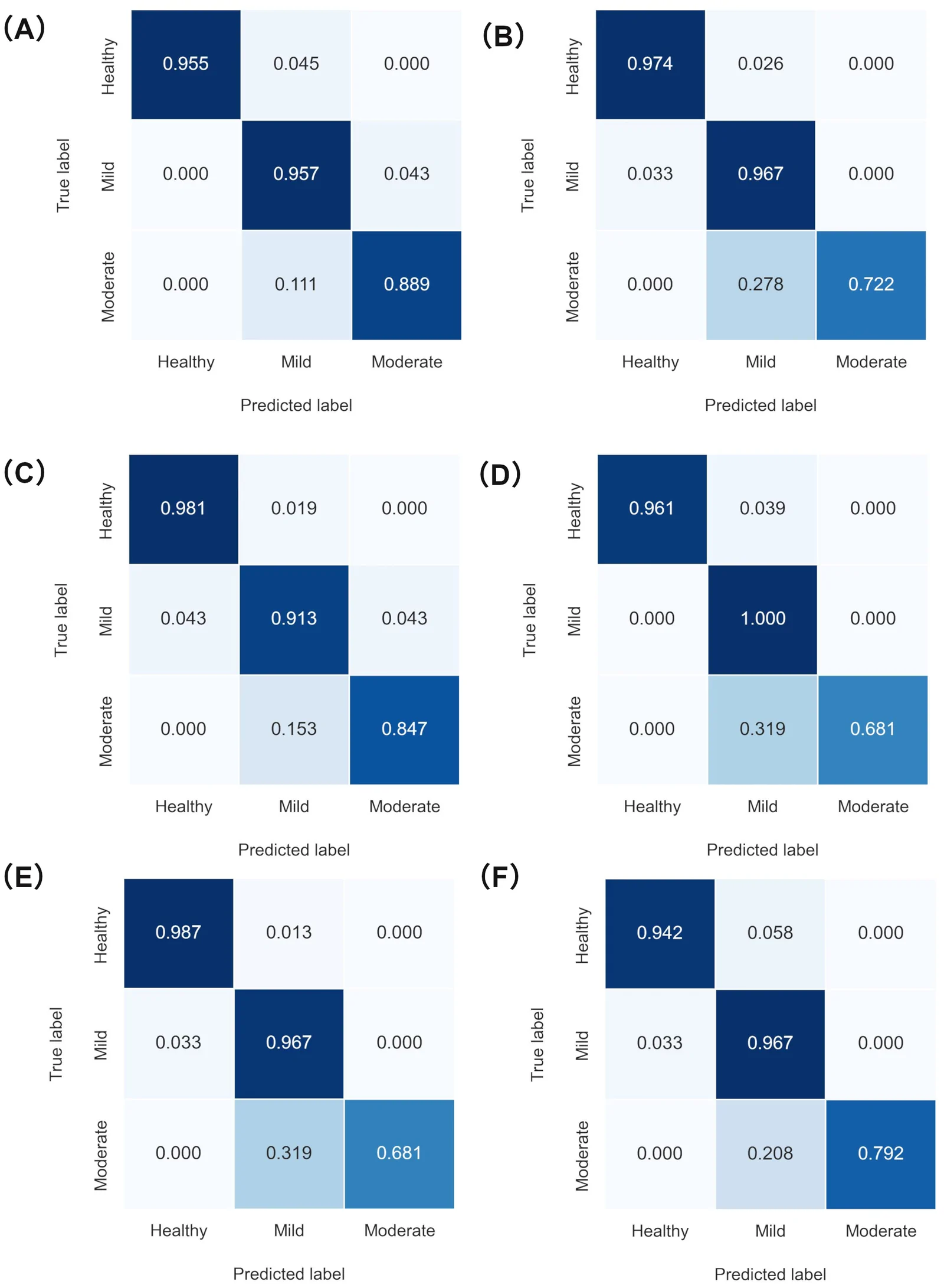
Confusion matrix of classification effect of each model. **(A)** LCRNet. **(B)** AlexNet. **(C)** GoogLeNet. **(D)** EfficientNet-B0. **(E)** EfficientNetV2. **(F)** ConvNeXt.

## 5 Discussion

This study integrated chlorophyll fluorescence imaging with deep learning to systematically investigate the response patterns of key fluorescence parameters to wheat powdery mildew stress and to construct an effective disease severity classification model, LCRNet. The results indicated that QYmax was a robust indicator for disease recognition. Furthermore, the newly developed LCRNet model achieved good performance in classifying fluorescence images (94.0% accuracy, 94.1% F1-score), providing an effective solution for wheat powdery mildew detection.

QYmax showed the strongest negative correlation with DS (r = -0.77), indicating that powdery mildew infection severely suppressed the function of photosystem II (PSII), a core unit of photosynthesis. Although QYmax demonstrated the strongest diagnostic efficacy, Rfd and Y(NO) still provided important complementary information. Their moderate correlation with DS and their highly significant mutual negative correlation (r = -0.83) collectively revealed an intense competitive relationship between photochemical energy conversion and non-regulated energy dissipation within PSII under stress. Particularly during severe infection stages, the sustained decline in QYmax coupled with a sharp rise in Y(NO) indicated that PSII might transition from reversible photoinhibition to irreversible damage. This severe functional degradation of PSII likely stemmed from impaired electron transport downstream of QA. This process led to excessive accumulation of reduced QA (QA^−^), elevating the excitation pressure at the reaction center and inducing excessive reactive oxygen species (ROS) production in the PSII reaction center. ROS attacked and degraded the D1 protein. If the *de novo* synthesis of D1 protein was insufficient to compensate for degradation losses at this stage, the PSII repair cycle became obstructed, ultimately triggering irreversible photoinhibition (Gururani et al., 2015). To ensure the robustness of the evaluation results and enhance the model’s interpretability, this study first conducted a comprehensive analysis of the model using a 5-fold cross-validation strategy and Grad-CAM visualization techniques. Based on this, a comparison with five mainstream deep learning models revealed that the LCRNet model achieved the best overall performance in the classification task (94.0% accuracy and recall, 94.4% precision, and an F1 score of 94.1%). Its high F1 score of 94.1% indicated that the model achieved a good balance between accurately identifying disease-infected areas (high precision) and comprehensively detecting actual infection sites (high recall). LCRNet’s performance advantages stemmed from the design of its core feature processing module. This module used the LSAF module to capture global context and expand the effective receptive field, while combining it with the CA module to achieve adaptive weighting of channel features. The synergy between these two components significantly enhanced feature discriminative power while keeping the number of parameters at 12.11M, thereby achieving an optimized balance between performance and computational efficiency. In contrast, classical models such as AlexNet lacked efficient attention and residual mechanisms, resulting in insufficient feature extraction capabilities and robustness. While models like EfficientNet-B0 and GoogLeNet had extremely low parameter counts (4.01M and 5.97M, respectively) and were better suited for edge deployment, their classification performance (accuracy of 90.9% and 93.1%) lagged behind that of LCRNet. Although models such as EfficientNet-V2 and ConvNeXt also achieved good performance (accuracy rates of 91.2% and 91.5%, respectively), none surpassed LCRNet. This demonstrated that, given the data characteristics of fluorescence images of wheat powdery mildew, LCRNet had a more suitable architecture for these fluorescence image data.

Although this study achieved preliminary results, several limitations remained to be addressed in future work. First, the dataset exhibited limited spatiotemporal coverage. The research only spanned part of the vegetative growth period. This study covered only part of the vegetative growth period, which may have overlooked critical physiological transition points. In addition, the limited diversity of cultivars and geographic regions may complicate model transfer and deployment in new environments. Second, despite efforts to control for it, the inherent variability of field conditions could still introduce noise into fluorescence measurements. Finally, the spatial heterogeneity of disease occurrence in the field presented a fundamental challenge to data consistency and model robustness. This was specifically reflected in the fluorescence parameter responses observed in this study. For instance, the fluorescence decay rate (Rfd) exhibited variability across plots, demonstrating markedly lower response stability compared to QYmax. This discrepancy likely stemmed from the coupled effects of multiple factors, including uneven lesion distribution, microclimate gradients between plants, and diverse background physiological states. Furthermore, an individual plot (e.g., Plot 1) exhibited unique fluorescence parameter patterns that deviated from the overall trend. Such anomalies might have arisen from variations in individual plant health, subtle differences in infection progression, or interactions between local environmental conditions and genetic background. Collectively, these observations demonstrated that complex spatial heterogeneity at the field scale, driven by both biotic and abiotic factors, significantly increased the difficulty of acquiring uniform, representative datasets. Future research should expand the dimensionality and scale of the data by collecting long-term time-series fluorescence images across multiple cultivars, geographic regions, and complete growth cycles; explore multimodal information fusion by integrating chlorophyll fluorescence data with other remote sensing sources, such as hyperspectral and thermal infrared imagery; and foster interdisciplinary innovation to facilitate the translation of this technology into practical precision agriculture applications.It should be noted that disease severity quantification in this study was performed using the Leaf Doctor software, which has been evaluated in previous studies for its reliability in assessing plant disease severity. Nevertheless, further studies specifically comparing the software’s segmentation performance with expert-annotated ground truth for chlorophyll fluorescence imagery of wheat powdery mildew would be valuable to further establish its applicability in this imaging modality.

## 6 Conclusions

This study innovatively integrated chlorophyll fluorescence imaging with deep learning algorithms to establish a technical framework spanning from the analysis of disease-induced physiological responses to intelligent phenotypic recognition, offering a novel approach to intelligent classification of wheat powdery mildew. The main contributions of this research were as follows:

1. This study elucidated the temporal response patterns of key chlorophyll fluorescence parameters to disease severity and revealed the correlation between critical fluorescence indicators (particularly QYmax) and disease severity, confirming QYmax as a robust indicator for disease response. Based on this, a dedicated wheat powdery mildew dataset based on QYmax fluorescence images was constructed, establishing a data foundation for algorithmic research.
2. This study proposed the LCRNet model for severity classification of wheat powdery mildew based on chlorophyll fluorescence images, whose core innovation lay in the design of a synergistic feature processing mechanism between the LSAF and CA modules, which achieved improved recognition by expanding the receptive field and enhancing inter-channel feature fusion.
3. This study validated, through extensive ablation and comparative experiments, LCRNet’s superiority in both recognition accuracy and parameter efficiency, demonstrating its role in advancing crop disease control toward intelligent and precision-oriented approaches.

This study demonstrated the significant potential of deeply integrating plant physiological phenotyping with artificial intelligence for disease severity grading and recognition. Furthermore,it provided crucial methodological support and technical insights for promoting the intelligent and precision-oriented green transformation of crop disease prevention and control systems.

## Data Availability

The original contributions presented in the study are included in the article/supplementary material. Further inquiries can be directed to the corresponding author.
